# T-Cell Large Granular Lymphocytic Leukemia with Extremely Rare Immunophenotype (CD4/CD8 Double-Positive) Followed by Multiple Myeloma Diagnosis

**DOI:** 10.1155/2020/8839144

**Published:** 2020-08-14

**Authors:** Dina Soliman, Sherin Sallam, Susanna Akiki, Deena Mudawi, Feryal Ibrahim

**Affiliations:** ^1^Department of Laboratory Medicine and Pathology, National Center for Cancer Care and Research, Hamad Medical Corporation, Doha, Qatar; ^2^Weill Cornell Medicine, Doha, Qatar; ^3^Department of Clinical Pathology, National Cancer Institute, Cairo University, Cairo, Egypt; ^4^Department of Hematology and Medical Oncology, National Center for Cancer Care and Research, Hamad Medical Corporation, Doha, Qatar

## Abstract

T-cell large granular lymphocytic leukemia is characterized by clonal expansion of a CD3^+^/CD57^+^ subpopulation, which are typically CD8^+^ positive cytotoxic T- cells, and can only be diagnosed if there is a persistent, greater than 6 months, elevation of LGL in the blood (usually 2–20 × 10^9^/L), in the absence of an identifiable cause. T-LGLL has been associated with reactive conditions such as autoimmune diseases and viral infections and has also been reported in association with hematologic and non-hematologic malignancies. We report a case of asymptomatic CD4/CD8 double-positive T-LGLL. Flow cytometry on peripheral blood revealed a subpopulation of CD4/CD8 double-positive T cells expressing CD57 and cTIA. Clonality was established by flow cytometric analysis of T-cell receptor *V*(*â*) region repertoire which showed that >70% of the cells failed to express any of the tested *V*(*â*) regions. Clonality was further confirmed by PCR with the detection of clonal TCR beta and TCR gamma gene rearrangements. Six months later, she presented with persistent lower back pain and diagnosed with IgG kappa multiple myeloma. CD4/CD8 double-positive T-large granular leukemia is the first case reported in the literature. This rare phenotype is either underreported or a truly rare clinical entity. More studies are warranted to characterize the pathogenesis and clinical characteristics of this group of patients and to further assess the relationship between multiple myeloma and T-LGLL as a cause-and-effect relationship or simply related to the time at which diagnosis has been made.

## 1. Case Presentation

A 54-year-old female, with no known chronic illnesses, was referred under the care of the hematology team after the incidental finding of thrombocytopenia and lymphocytosis in her routine laboratory workup. The patient had no abnormal bleeding or ecchymosis. She also denied any fever, weight loss, or skin rash. At that time, her platelet count was 65,000/*µ*L.

Complete blood count (CBC) revealed mild leukocytosis (WBC count of 12,600 × 10^3^/uL; *n* : 4–10) with absolute lymphocytosis (7.3 × 10^3^/uL) and no neutropenia. Her initial workup showed high total protein at 95 gm/L (66–78), normal albumin at 4.3 g/dL, and normal beta-2-microglobulin (2.09 mg/L) with mildly elevated LDH at 258 U/L (135–214). Her calcium level was normal (2.36 mmol/L), with normal electrolytes and normal liver and kidney function tests.

**Figure 1:**
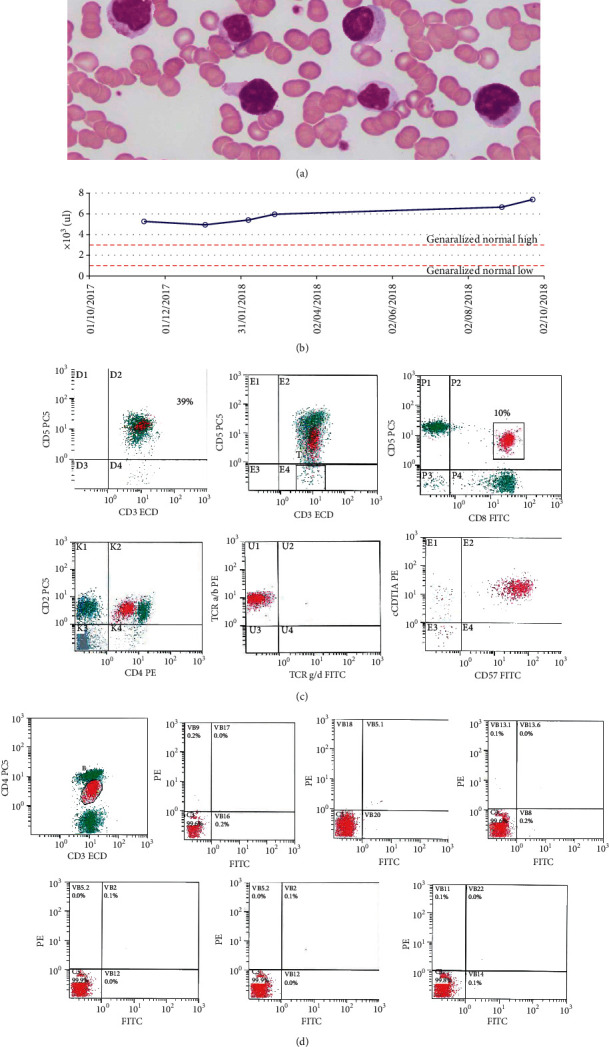
Peripheral blood smear (Wright's stain, 100x) shows increased large granular lymphocytes, some of which showed irregular nuclear contours (a). Lymphocytosis is persistent and slowly progressive for more than 11 months of duration (b). Flow cytometry done on the PB (c) showing 37% T cells, including a subpopulation of CD4/CD8 double-positive T cells (10%) expressing TCR alpha/beta, CD57, and cTIA, and a showing dim expression of CD4 and CD7. FCM on PB for T-cell receptor *V*(*β*) region repertoire on CD4/CD8 double-positive population shows more than 70% of the cells fails to express any of the tested *V*(*β*) regions, suggestive of clonal population of T-LGLL (CD3^+^/CD4^+^/CD8^+^/CD57^+^) (d).

The peripheral blood (PB) smear showed mild anemia with increased rouleaux formation. There was lymphocytosis composed of small mature-looking lymphocytes mixed with some large granular forms, some of which showed irregular nuclear contours (Figure 1(a)). Lymphocytosis is persistent and slowly progressive for more than 11 months of duration (Figure 1(b)). Flow cytometry done on the PB (Figure 1(c)) showed 37% T cells, including a subpopulation of CD57-positive cells (NK-T cells) comprising approximately 17%. There was a subpopulation of CD4/CD8 double-positive T cells (10%) expressing CD57 and cTIA and showing a dim expression of CD4 and CD7. Flow cytometric analysis of T-cell receptor *V*(*â*) region repertoire performed on CD4/CD8 double-positive population (on PB) (Figure 1(d)) showed that more than 70% of the cells failed to express any of the tested *V*(*â*) regions, a finding that is suggestive of T-cell clonality.

Six months after her initial presentation, the patient presented with persistent lower back pain. On physical examination, there was tenderness at the lumbosacral junction with no distal nerve deficit. MRI showed an old fracture of the L1 body and a large Schmorl's node-like lesions within the superior end plates L3 and L4. CT thorax, abdomen, and pelvis showed evidence of an infiltrative bone marrow (BM) disease.

MRI whole spine showed heterogeneous marrow signal pattern of the visualized bones with multiple osseous lesions possibly indicative of an infiltrative myelomatous deposits. FDG PET showed multiple small lytic lesions.

Serum protein electrophoresis was performed as part of the myeloma workup and revealed hypogammaglobulinemia and a monoclonal band typed as IgG kappa 28.8 g/l in size. The kappa : lambda ratio was 43.595 with the kappa free light chains at 352.68 mg/L and the lambda free light chains at 8.09 mg/L. 24-hour urinary protein was negative for Bence Jones protein.

**Figure 2:**
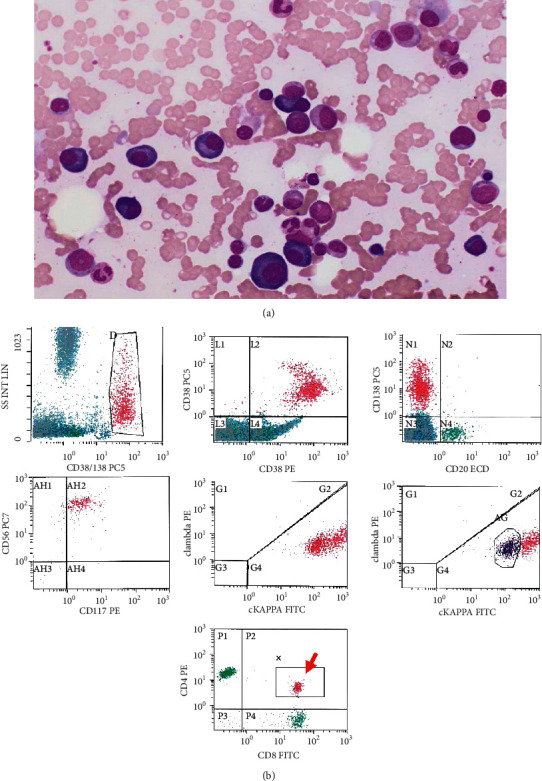
BM aspirate smear (Wright's stain, 100x) revealing ∼18% myeloma plasma cells with a few atypical binucleated forms (a). FCM analysis on BM aspirate (b) showing a population of monotypic plasma cells ∼5% expressing CD45, CD38, and CD138 with cytoplasmic kappa light chain restriction and aberrant expression of CD56 and CD117. The plasma cells show no significant expression of CD20. LGLs included the same population of CD4/CD8 double-positive cells (∼2%) (red arrow) showing a dimmer expression of CD4 and CD7.

BM examination was performed, and PB showed persistent lymphocytosis with no neutropenia. BM aspirate smear (Figure 2(a)) revealed ∼18% myeloma plasma cells with a few atypical binucleate forms and rare immature forms with plasmablastic morphology. BM biopsy was infiltrated by many myeloma plasma cells with kappa light chain restriction. BM involvement by myeloma cells was uneven, roughly estimated by 30–60% of cellularity. Flow cytometry on BM aspirate (Figure 2(b)) showed a population of monotypic plasma cells ∼5% expressing CD45, CD38, and CD138 with cytoplasmic kappa light chain restriction and aberrant expression of CD56 and CD117. The plasma cells were negative for CD19 with no significant expression of CD20. LGL included a population of CD4/CD8 double-positive cells (∼2% of total cells) showing a dimmer expression of CD4 and CD7 (similar to the population detected earlier on peripheral blood) (Figure 2(b), red arrow). Flow cytometric analysis of T-cell receptor *V(â)* region repertoire on CD4/CD8 double-positive population (on PB) was repeated and showed persistent clonal population.

Cytogenetic analysis revealed a normal karyotype. FISH testing for the following genes was normal: RB1/CEP10 : 13q14, TP53/CEP17 : 17p13.1/17p11.1-q11.1, and IGH : 14q32.

This concluded a diagnosis of plasma cell myeloma associated with a persistent increase in clonal large granular lymphocytes (CD4/CD8-positive) indicating T-large granular lymphocytic leukemia (T-LGLL).

T-cell receptor (TCR) gene rearrangement molecular genetics studies were performed and confirmed the presence of clonal TCR beta and TCR gamma gene rearrangements: TCR beta DBJ-C : clonal (175 bp and 305 bp) and TCR gamma VGJ-B : clonal (204 bp).

The patient was started on bortezomib-based therapy regimen, VRd (Velcade® one dose (cycle 1, day 1), lenalidomide 25 mg for 3 days, and dexamethasone pulse for 4 days), followed by stem cell mobilization and high-dose melphalan and autologous stem cell transplantation (tandem).

## 2. Discussion

This is a rather unusual case of asymptomatic T-LGLL with a unique immunophenotype incidentally discovered upon workup for lymphocytosis, followed by multiple myeloma diagnosed 6 months after T-LGLL diagnosis.

T-cell large granular lymphocytic leukemia is characterized by the clonal expansion of a CD3^+^/CD57^+^ subpopulation, referred to as large granular lymphocytes which are morphologically distinct, larger than small mature lymphocytes with mature nuclear chromatin and moderate-to-abundant cytoplasm and fine or course azurophilic granules. T-LGLL is typically characterized by CD8+ positive cytotoxic T cells and characterized clinically by neutropenia, anemia, and/or thrombocytopenia with modest persistent lymphocytosis [1].

According to the most recent WHO classification of hematologic disorders (2016), T-LGLL can only be diagnosed if there is a persistent, greater than 6 months, elevation of LGL in the blood (usually 2–20 × 10^9^/L), in the absence of an identifiable cause. It is usually a disorder of mature CD2+, CD3+, CD8+, CD57+, and alpha-beta TCR-positive cytotoxic T cells. CD4+/alpha-beta TCR-positive cases are uncommon variants. Gamma-delta TCR-positive cases, with 60 percent of them expressing CD8 and the remainder being CD4/CD8-negative, are also uncommon variants. T-LGLL that is CD4/CD8 double-positive is not documented [2].

T-LGLL is a relatively rare malignancy and accounts for 2–5% of chronic lymphoproliferative diseases in the US [1]. It appears more frequently in the elderly, with a median age of diagnosis at 60 years and median survival of more than 10 years [3]. It has been hypothesized that T-LGLL arises in a setting of sustained immune stimulation which could explain the frequent association of T-LGLL with autoimmune disorders, including rheumatoid arthritis and inclusion body myositis [4–6].

CD4/CD8 dual-positive T cells are a developmental stage of T-cell maturation/differentiation within the thymus. The interaction of T-cell receptor with antigens bound to major histocompatibility complex (MHC) molecules causes the dual-positive T cell to commit to either CD4 (helper and regulatory) or CD8 (cytotoxic and suppressor). Besides mature single-positive CD4+ or CD8+ lymphocytes, extrathymic CD4+/CD8+ double-positive T cells are present in a small percentage in the peripheral blood of healthy humans [7].

On the contrary, multiple myeloma (MM) is a different hematologic neoplasm of B-cell lineage, characterized by the clonal proliferation of plasma cells in the bone marrow resulting in accumulation of monoclonal immunoglobulin. It usually presents with hypercalcemia, renal failure, anemia, and bony lesions [8]. There is a well-documented association between T-LGLL leukemia and B-cell neoplasms, in general, and plasma cell neoplasms, in particular. Upon review of the literature, a total of 42 patients with a diagnosis of T-LGLL and a plasma cell neoplasm were found [9–13]. The majority of these cases had a concomitant diagnosis of both T-LGLL and a plasma cell neoplasm or had a plasma cell neoplasm diagnosis followed by T-LGLL. Interestingly, in the largest case series of patients diagnosed with T-LGL and a plasma cell disorder by Sidiqi et al., all reported 22 cases, who had T-LGLs, showed the classic immunophenotype (CD8+ positive cells) [12]. Not a single case of CD4/CD8 dual-positive T-LGLL was reported.

The detection of an expanded population of LGL after MM treatment was previously described. In one study by Hashiguchi et al., a patient with MM received lenalidomide as part of a clinical trial. They noted an expansion in peripheral LGL from 8% prior to initiating lenalidomide to 25–40% after. The study proposed a beneficial immunomodulatory effect of increasing NK/T cells in patients with MM [14]. This is particularly important because MM patients have been shown to demonstrate a deficit in NK/T-cell counts and activity [15]. In one study, none of the 12 patients diagnosed with T-LGLL and MGUS progressed to MM [13]. This has raised the possibility that the T-LGL clonal expansion represents an immune response to MGUS and suggests a potential role in tumor surveillance [16].

In addition to posttreatment proliferation of LGL, there have also been descriptions of proliferation after bone marrow transplant for refractory MM. Wolniak et al. noted the proliferation of LGL after BM transplant in 20 patients with MM and concluded that this proliferation is a reactive response to the autologous BM transplant [17]. Based on the studies by Hashiguchi et al. and Wolniak et al., and the other studies that described the development of MM in the setting of T-cell LGL or vice versa, it is clear that the relationship between the two disorders is complicated and needs further studies [14, 17].

It has been shown that CD4-positive T-LGLL and CD4/CD8 double-negative T-LGLL are rarely reported in the literature [18, 19]. CD4/CD8 double-positive T-LGLL (as the case reported here) is even more rare. On a similar note, Mizuki et al. studied 8 cases of CD4+/CD8+ chronic T-lymphoproliferative disorders and have found that the dual-positive T cells expressed IL-4 mRNA and produced large amounts of IL-4, suggesting that they are derived from peripheral CD4+ cells rather than immature T cells that escaped the thymus. Of note, none of these cases were T-LGLL [20]. On the other hand, for other types of leukemia such as the adult T-cell leukemia/lymphoma associated with HTLV1, the dual-positive phenotype has been extensively described [21–24]. It also seems that the expansion of clonal CD4/CD8 double-positive T-LGL, although fulfilling WHO diagnostic criteria for T-LGLL, has an even more indolent clinical course than the typical CD8+ T-LGL leukemia as our patient's case lacked the typical clinical features such as neutropenia and splenomegaly.

## 3. Conclusion

To our knowledge, this the first case report of T-LGLL with the extremely rare phenotype (CD4/CD8 double-positive) associated with multiple myeloma. This might suggest that there is either an underestimation of this rare phenotype in large granular lymphocytic leukemia or, indeed, a rare entity that requires further studies. Another distinguishing feature is that the diagnosis of LGL was made before establishing MM, and is again another unusual scenario, when compared with previous reports. Whether the relationship between the two hematologic neoplasms is a cause-and-effect relationship or simply a time to diagnose issue is yet to be investigated. Furthermore, uncontrolled expansion of clonal T cells (LGL) as one of the mechanisms of host immune response to multiple myeloma is also a possibility.

According to published data on patients who had a concomitant PCM and T-LGL, therapy directed towards the T-LGL component was needed in few patients, and the therapy consisted of cyclophosphamide and prednisone with good response and resolution of the cytopenias in most patients.

## Data Availability

The data supporting the findings of this study are available from the corresponding author upon reasonable request.
